# The complete chloroplast genome of the medicinal plant *Angelica decursiva* (Apiaceae) in Peucedani Radix

**DOI:** 10.1080/23802359.2016.1155089

**Published:** 2016-03-28

**Authors:** Sun A. Choi, Ye Ji Kim, Woo Kyu Lee, Kyu Yeob Kim, Jong Hwan Kim, Rack Seon Seong

**Affiliations:** a Herbal Medicine Research Division, National Institute of Food and Drug Safety Evaluation, Ministry of Food and Drug Safety, Cheongju-si, Republic of Korea;; b Herbal Medicine Policy Division, Ministry of Food and Drug Safety, Cheongju-si, Republic of Korea

**Keywords:** *Angelica decursiva*, Apiaceae, chloroplast genome, next-generation sequencing, Peucedani Radix

## Abstract

*Angelica decursiva* (Miquel) Franchet & Savatier (Apiaceae) has been used as a significant medicinal plant in East Asia. We determined its complete chloroplast genome for the first time in this study. The complete chloroplast was circularized and had a typical quadripartite structure genome of 146 719 bp long including the large single copy region (LSC) of 93 256 bp, the small single copy region (SSC) of 17 497 bp and duplicated inverted regions (IRs) of 17 983 bp each. The total GC content was 37.56% and for the four structures it was 35.98% (LSC), 31.06% (SSC), and 44.83% (for each IR). There were a total of 113 genes, comprising four rRNAs, 29 tRNAs and 80 protein coding genes. In the phylogenetic analysis, *A. decursiva* was grouped with *Seseli montanum.* This study may contribute to authenticating the plant’s correct use as medicine for health and provide an important genetic resource for phylogeny with related species.

Peucedani Radix has been used in significant traditional herbal medicines in East Asia that treat colds, coughs and fevers from wind-heat (Kong et al. 1996; Menglan et al. 2005). Peucedani Radix is derived from the dried roots of *Peucedanum praeruptorum* Dunn or *Angelica decursiva* (Miquel) Franchet and Savatier, in Korea (KFDA, 2012), Japan (The Ministry of Health, Labour and Welfare 2011) and Taiwan (Ministry of Health and Welfare 2005), while only *P. praeruptorum* is approved in China (Pharmacopoeia Commission of the People's Republic of China 2010). To prevent confusion owing to the differences in herbal medicinal regulations within various countries, we examined the chloroplast genome importantly used to understand the identification, phylogeny, genetic population and evolution for plant studies using next-generation sequencing (NGS). We generated the first complete chloroplast genome of *A. decursiva*, one of the sources of Peucedani Radix, which will be helpful for identifying herbal medicines correctly.

The plant materials for genomic sequencing were collected from cultivated *A. decursiva* in the National Center for Herbal Medicine Resources, the NIFDS and the MFDS of Korea in 2013. The extracted genomic DNA was constructed using an Illumina paired-end (PE) library and sequenced using the Illumina Hiseq 2000 instrument (Illumina, San Diego, CA). The PE reads were assembled by CLC genome assembler (ver. 4.06 beta, CLC Inc, Rarhus, Denmark). Gene annotation and structure analysis were conducted by DOGMA (Wyman et al. 2004) and manually corrected on BLAST at PHYZEN Inc. (Seoul, Korea). The complete assembled genome sequence was deposited in GenBank under accession no. KT781591.

The complete chloroplast genome of *A. decursiva* was a circular molecule with 146 719 bp in length, which showed typical organization. The genome was divided into four structures: the large single copy region (LSC) of 93 256 bp, the small single copy region (SSC) of 17 497 bp and two copies of inverted regions (IRs) of 17 983 bp each. The total GC content of *A. decursiva* was 37.56%, with 35.98% for the LSC, 31.06% for the SSC and 44.83% for each IR. The genome contained four rRNAs, 29 tRNAs and 80 protein coding genes, for a total of 113 genes. The tRNA coding genes were widely distributed in the genome, with 22 in the LSC, one in the SSC and six in each IR, while the rRNA coding genes were only in the IRs. Each duplicated IR region involved six tRNAs (*trnA*^(UGC)^, *trnI*^(GAU)^, *trnL*^(CAA)^, *trnN*^(GUU)^, *trnR*^(ACG)^ and *trnV*^(GAC)^), four rRNAs (*rrn4.5*, *rrn5*, *rrn16* and *rrn23*) and eight protein coding genes (*ndhB*, *orf42*, *orf56*, *rps7*, *rps12*, *ycf68*, *ycf1* and *ycf2*).

A total of 11 taxa, comprising *A. decursiva* in Apiaceae and the closely related family Araliacae as outgroups, were aligned using MAFFT (http://mafft.cbrc.jp/alignment/software/). To analyze phylogeny, a maximum likelihood (ML) tree was generated by general time reversible parameters based on gamma distribution and 1000 replications at GARLI Web Services (www.molecularevolution.org). In the ML phylogenetic tree, Apiaceae was closely related to family Araliaceae and *A. decursiva* was closely related with *Seseli montanum* (Figure 1).

**Figure 1.**
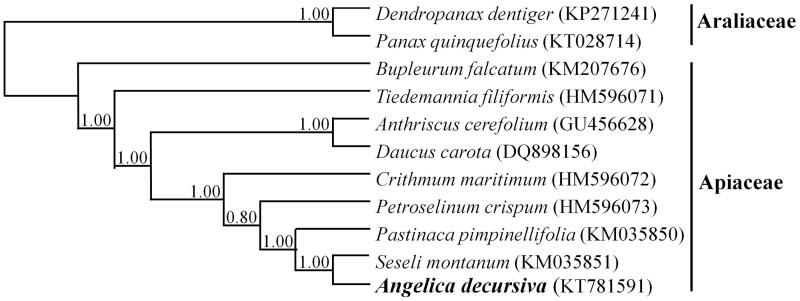
Maximum likelihood analysis of *Angelica decursiva* with related species in Apiaceae and outgroup species in Araliaceae, based on the complete chloroplast genome sequences. Numbers on branches indicate the bootstrap values.

The first reported chloroplast genome of *A. decursiva* will support estimation for the authentication of Peucedani Radix in each country and the prevention of adulterations. It could also be the basic data for understanding the phylogenetic relationships in the genus *Angelica* and the family Apiaceae.
